# A Comparative Study of Cast‐Tape, Freeze, and Oven Drying on the Physicochemical and Bioactive Properties of Red Cabbage Microgreen (*Brassica oleracea* var. *capitata* f. *rubra*) Foam Powders

**DOI:** 10.1111/1750-3841.70959

**Published:** 2026-02-28

**Authors:** Islaine De Jesus Silva, Mônica Silva De Jesus, Hannah Caroline Santos Araujo, Regina Santiago Campos Nascimento, Maria Terezinha Santos Leite Neta, João Paulo Natalino De Sá, Simone Mazzutti, Frederico Alberto De Oliveira, Marcelo Augusto Gutierrez Carnelossi, Angelise Durigon

**Affiliations:** ^1^ Food Technology Department Federal University of Sergipe São Cristóvão Sergipe Brazil; ^2^ Laboratory of Flavor and Chromatographic Analysis, PROCTA Federal University of Sergipe São Cristóvão Sergipe Brazil; ^3^ Sertão Agroindustry Department Federal University of Sergipe Nossa Senhora da Glória Sergipe Brazil; ^4^ Education in Agrarian and Earth Science Department Federal University of Sergipe Nossa Senhora da Glória Sergipe Brazil

## Abstract

The aim was to produce red cabbage microgreen foams and dry them by cast‐tape drying (CTD), freeze‐drying (FD), and oven drying (OD) to obtain powder and characterize them. The foam composition was water (74.7%), microgreens (14.2%), emulsifier (6.5%), pre‐gelatinized starch (2.8%), and maltodextrin (1.8%). Drying conditions were CTD (98°C, 2–3 mm thick), FD (0.14 mbar, −58°C, for 48 h), and OD (70°C, 2–3 mm thick). Drying times were 32 min for CTD, 2880 min for FD, and 150 min for OD (moisture <0.1 g/g). The foam spreads easily in the CTD, enabling quick drying, whereas OD and FD processes take longer. The FD powder exhibited higher levels of phenolics, flavonoids, anthocyanins, and antioxidant activity despite high water activity and a greener coloration. The Fourier transform infrared spectroscopy (FTIR) spectrum of the powders, especially FD and CTD, indicated the presence of bioactive compounds (flavonoid compounds), carbohydrates (starch, maltodextrin, cellulose, and hemicellulose), and fatty acids (esterified carboxyl groups). CTD enabled rapid and scalable processing but led to greater bioactive degradation due to temperature and light exposure, whereas OD provided an intermediate balance between process efficiency and compound preservation. Foam formulation resulted in low moisture and water activity in CTD and OD powders. Overall, the findings highlight trade‐offs between drying efficiency and nutritional quality, supporting the use of microgreen powders in value‐added food formulations.

**Practical Applications:**

Powdered microgreens are versatile and easy to incorporate into most meals and beverages as a neutral nutritional booster or even a flavor enhancer. The foam red cabbage microgreens dehydrated by freeze‐drying, cast‐tape drying, and hot‐air drying produce purple cabbage powders with interesting physicochemical properties and significant amounts of bioactive compounds, such as phenolics and anthocyanins, particularly in those obtained by freeze‐drying. These characteristics highlight the great potential for utilization and possible applications of these products in various fields of food, nutrition, and pharmaceuticals.

## 1 Introduction

The global relevance of plant‐derived products is rising with increasing consumer interest in plant proteins, fibers, vitamins, minerals, and bioactive compounds (Bryant 2022). Given the increasing demand for plant‐based products, creating stable, value‐added, plant‐based products is now a priority for research and the food industry. Promising options include sprouted shoots, microgreen cultivation, and baby leaf production.

Microgreens, nutrient‐rich seedlings, are recognized as functional foods and consist of immature, delicate vegetables grown from the seeds of vegetables, herbs, legumes, and wild species (Lone et al. 2024; Sanyukta et al. 2023). Their cultivation favors both the environment and economic sustainability (Lone et al. 2024). They are cultivated in substrates or hydroponically, under light, and harvested 10–28 days after emergence, when the cotyledon leaves are fully expanded, and the first true leaves may or may not have appeared (Xiao et al. 2016).

Microgreens from *Brassicaceae* species such as red cabbage (*Brassica oleracea* var. *capitata* f. *rubra*) are rich in micronutrients (N, P, K, Ca, Mg, Mn, and Zn) and bioactive compounds, including ascorbic acid (406–628 mg/100 g), quercetin (QE) (396–407 mg/100 g), and anthocyanins (240–300 mg/100 g) (De Freitas 2020). Despite their nutritional, functional, and sensory benefits, microgreens have limitations like low productivity, rapid senescence, and short postharvest shelf life, which hinder their commercial production (Chandra et al. 2012; Kou et al. 2013). Transforming microgreens into powder is a promising strategy to extend shelf life, facilitate handling and transportation, and expand their use as functional food ingredients (Bhasin et al. Rasane et al. 2023; Mansouri et al. 2024; John et al. 2025; Jauregui et al. 2025).

Drying methods, such as freeze‐drying (FD), cast‐tape drying (CTD), and oven drying (OD), can be used to produce powdered foods, and this study focuses on foam‐mat drying applied within these techniques to optimize the process. Red cabbage microgreens were selected as a model system because of their high anthocyanin and phenolic contents, which are highly sensitive to processing conditions and thus suitable indicators for assessing the effects of drying methods.

Foam‐mat drying is an interesting method for dehydrating microgreens, as it uses milder temperatures ideal for heat‐sensitive compounds. The process involves producing stable foam using foaming agents and spreading this foam on a heated surface for drying (Seerangurayar et al. 2017; Hardy and Jideani 2017). Typically conducted in convective dryers (OD), although it can be carried out using other methods if it is done on the surface. Foam‐mat drying allows gentle dehydration while improving drying efficiency and powder quality (Seerangurayar et al. 2017).

FD is recommended for heat‐sensitive products and effectively preserves food flavor and aroma due to the absence of air and processing temperatures (Garcia‐Amezquita et al. 2016). FD was included in this study as a reference method due to its well‐established ability to preserve bioactive compounds (Oyinloye and Yoon 2020).

CTD has been extensively employed in the producing of powdered foods due to its great versatility. It is suitable for drying fruits, vegetables, and herbs due to its moderate temperatures and reduced drying times (Nindo et al. 2003; Santos et al. 2022; I. Tontul et al. 2018). The food temperature remains around 20–25°C lower than the heating source temperature (between 50°C and 95°C) for most of the process. Thin layers (2–3 mm) of the food are spread on the support (fiberglass coated with Teflon or polyester Mylar film) heated with water or steam (Durigon et al. 2018). CTD was selected as an emerging alternative due to its shorter drying times, moderate product temperatures, and potential applicability on an industrial scale.

Recent patent filings further reinforce the growing industrial interest in microgreen powders as functional food ingredients and the need for research on drying, powder characterization, and bioactive compound preservation for industrial applications. In 2024, Bhasin et al. (2024) patented an instant soup mix containing tomato powder, corn flour, black pepper, powdered red cabbage microgreens, powdered *Raphanus sativus* leaves, salt, and sugar. In this formulation, microgreen powders were used due to their potential to enhance the nutritional and functional value of convenience foods. Similarly, a 2025 patent by Rasane et al. (2023) describes an instant tea mix containing tea extract powder, microgreen extract powder, sucrose, milk solids, maltodextrin, and calcium carbonate, highlighting microgreen extracts’ potential for nutritional and functional enhancement in powdered beverages. The patent “A Process for Microgreens Based Powder” describes producing microgreen powders using spray, freeze, and drum drying. It also highlights their use as functional ingredients in foods, cosmetics, and pharmaceuticals (Rasane et al. 2021).

Recent studies have demonstrated the technological and functional potential of microgreen powder for food applications. Mansouri et al. (2024) demonstrated that sunflower microgreen powder (dried by cabinet drier at 50°C, 1.5 m/s) can be incorporated into gluten‐free cakes. The powder exhibited high protein (38.4% ± 0.3%), fiber (8.1% ± 0.4%), phenolics (39.34 ± 0.7 mg GAE/g), and antioxidant activity (AA) (87.36% ± 1.8% DPPH) while influencing texture and color. Freitas et al. (2024) obtained beet microgreen (*Beta vulgaris*) powders by CTD in 12–24 min versus 14–18 h for FD. In particular, the CTDF2 formulation (78.3% water + 13% microgreens + 4.4% pre‐gelatinized starch + 4.3% Emustab) showed lower moisture (0.09 g/g) and water activity (0.25) and stood out for its good flowability and low cohesiveness. However, a 50% loss of betalains was reported when compared to FD powder. All powders contained 4.0%–6.3% protein, highlighting their potential as a nutritious ingredient in plant‐based foods. Gunjal et al. (2025) studied convective drying of Sango radish microgreens (*R. sativus* L. var. *sango)* at temperatures of 40°C, 50°C, and 60°C and tray densities (0.057–0.113 g/cm^2^). Drying at 50°C with 0.057 g/cm^2^ retained the most anthocyanins and phenolics and showed the highest AA (DPPH, FRAP). Higher temperatures lowered water activity, improving microbial stability but increasing loss of thermolabile compounds. Jauregui et al. (2025) obtained radish microgreens (*R. sativus* L.) powders by hot air drying (45°C, 65°C, and 95°C). Drying at 95°C preserved total phenolics like FD, though glucoraphenin decreased by 21%. Anthocyanins were highly heat‐sensitive, showing reductions of about 49% across all temperatures, whereas vitamin C losses reached up to 63%, reinforcing the impact of drying conditions on thermolabile compounds. John et al. (2025) highlighted chia microgreen powder (CMP) as an innovative ingredient for wheat pasta fortification. Characterized by high protein content (34.7%), essential minerals (e.g., phosphorus 2070 mg/100 g; calcium 1840 mg/100 g), and total phenolics (13.63 mg GAE/g).

Despite the increasing number of studies on microgreens, the literature has primarily focused on their fresh form or on single drying methods, providing limited information on the comparative effects of different dehydration technologies on microgreen powder quality. Specifically, studies integrating foam‐mat drying with comprehensive physicochemical and bioactive compound characterization remain scarce. To the best of our knowledge, no comparative evaluations of FD, OD, and CTD applied to red cabbage microgreen foam have been reported.

This gap is relevant given the growing interest of the industry in stable and functional plant‐based ingredients. Using red cabbage microgreens as a model and comparing drying technologies with different properties, this study advances microgreen processing and provides insights for developing powders for food industry applications. Therefore, this study aimed to produce red cabbage microgreen foam and dry it by FD, OD, and CTD, followed by the characterization of the resulting powders in terms of physicochemical properties and bioactive compound content.

## 2 Materials and Methods

### 2.1 Material

The seeds (ISLA) of red cabbage *Krishna* microgreens, pesticide‐free and specifically intended for young harvest, were acquired on the company's website with an indicative germination rate of 95% and purity of 99.8%. The emulsifier Emustab (Selecta, Duas Rodas), maltodextrin, and pre‐gelatinized starch (DiLucca) were purchased from a local market in Aracaju, SE.

### 2.2 Obtaining Microgreens

The seeds were sanitized in a sodium hypochlorite solution before germination. Then, a dormancy break of 6 h was applied using cold water at 10°C. Subsequently, the seeds were immersed in a 10% hydrogen peroxide solution for 30 min for disinfection. The coconut fiber substrate, spread evenly in a perforated bottom tray with a 1.5 cm layer, was also sanitized using 10% hydrogen peroxide. Finally, the substrate was hydrated with enough filtered water to facilitate water circulation during irrigation.

**FIGURE 1:**
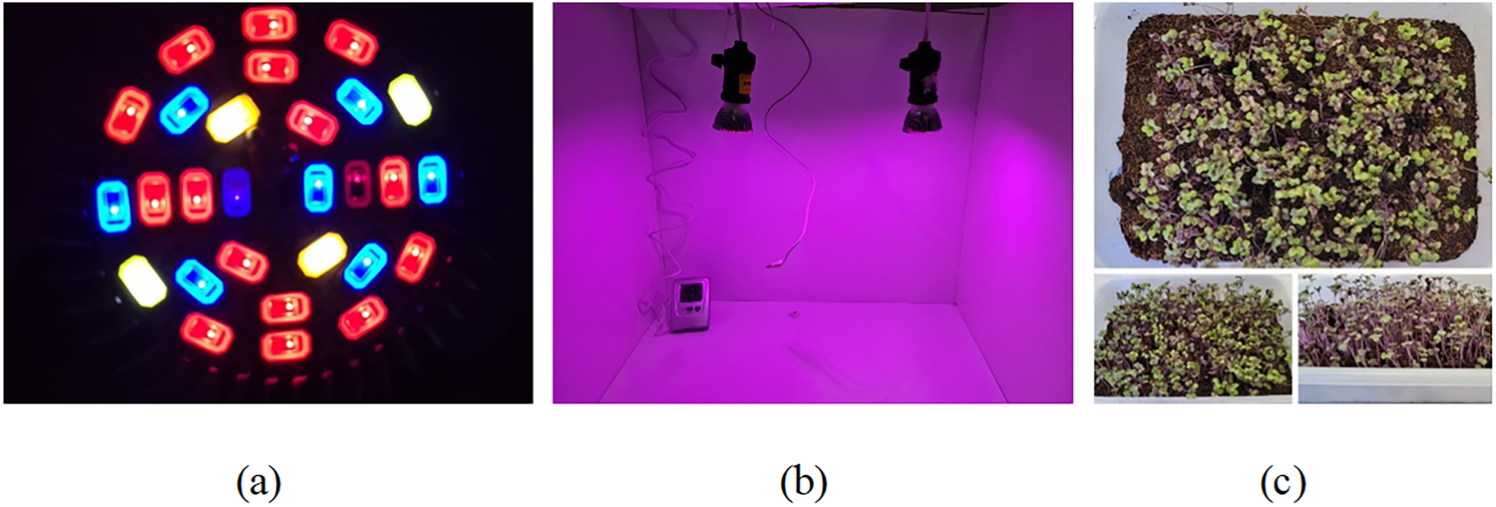
(a) LED lamp used to provide photo periods during the germination of microgreens, (b) germination chamber with temperature and relative humidity control, and (c) germination of red cabbage microgreen seeds.

The seeds were deposited on the substrate, covered with a 0.5‐cm layer of substrate, and remained in the dark for a period of 3 days until the first radicles appeared. Figure 1 shows photographs of seed germination for sprout production and the lighting system used during the photoperiod. Germination was conducted with 8 h photoperiods starting from the second day of germination and maintained for an additional 4 days, when the first seedling emerged, until the seventh day, when the microgreens were harvested. The photoperiod was facilitated with two LED lamps (LED grow indoor light—28 W) specifically designed for plant germination (De Freitas 2020). Each lamp contains 57.1% red bulbs, 28.6% blue bulbs, and 14.3% yellow bulbs (Figure 1a). This lamp setup creates a lilac‐colored environment and was strategically positioned to ensure uniform light intensity within the chamber (Figure 1b). LED lamp colors were selected on the basis of data showing the effectiveness of blue light (400–500 nm) and red light (660 nm). Irrigation was conducted three times a day at regular intervals, ensuring the substrate remained adequately moistened. The environment was maintained at temperature of 25.5°C ± 3°C and a relative humidity of 66% ± 4%. The germination rate was 79%, with an average length (hypocotyl + cotyledon) of 4.8 ± 0.9 cm (Figure 1c).

The foam of the microgreens was carried out due to the delicacy of their physical structure. The microgreen foam underwent the drying process through CTD, FD, and OD. For this, the harvested microgreens were subjected to a freezing process in an ultra‐freezer (SANYO, MDF‐U73VC) at −75°C ± 5°C, with the aim of their subsequent use in the formulations.

### 2.3 Preparation of the Formulations

Preliminary tests were conducted using different concentrations of emulsifier, pre‐gelatinized starch, maltodextrin, and water to determine the formulation for drying. The formulation choice was based on achieving the highest foam stability (<92%) within 120 min (Barmore 1934). Thus, the formulation was prepared with 74.7% of water, 14.2% of red cabbage microgreens, 6.5% of stabilizing emulsifier, 2.8% g of pre‐gelatinized starch, and 1.8% g of maltodextrin. These ingredients were placed in a container and mixed (Oster, FPSTHB2610R‐017) for 1 min to obtain a homogeneous mixture. The formulation was characterized for its soluble solids content in refractometer (Hanna, HI 96801, Rhode), moisture using a moisture analyzer balance (SHIMADZU, MOC63u), and pH using a digital pH meter (Quimis, model Q400MT) in triplicate, following (AOAC 2005) guidelines.

### 2.4 Drying Processes

#### 2.4.1 Cast‐Tape Drying

**FIGURE 2:**
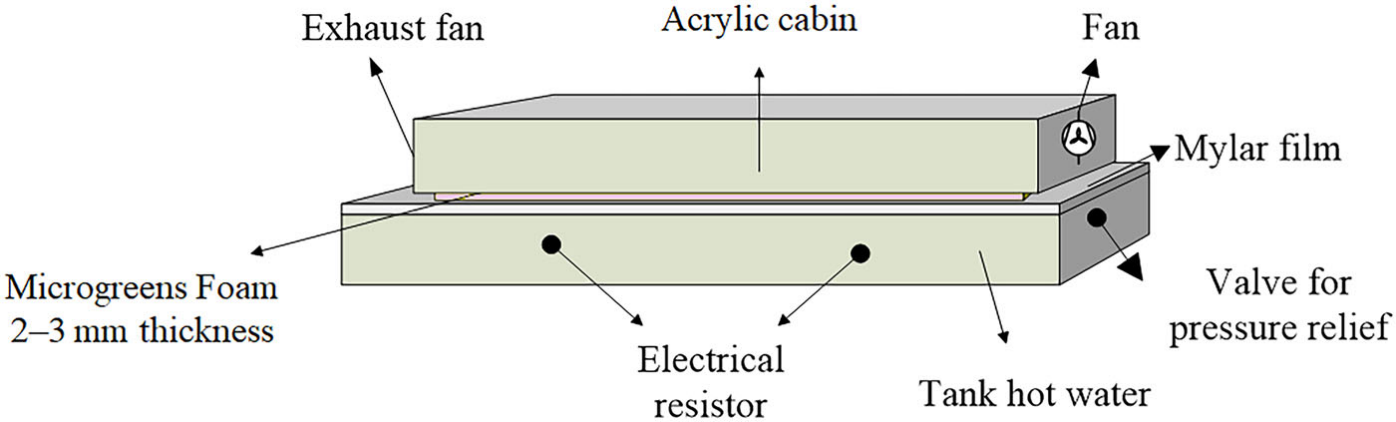
Foam is composed of microgreens of red cabbage, pre‐gelatinized starch, maltodextrin, water, and emulsifier.

The experiment consists of a tank (0.4 m x 0.2 m x 0.08 m) partially filled with water heated using electric resistors with a power of 2000 W (AGRATTO, FM‐01) (Figure 2). A 0.25 mm thick polyester film (Mylar Type D, DuPont) is fixed to the top of the tank (Da Silva et al. 2022). The lower side of the film is in contact with the water vapor generated from heating the water (water temperature of 98°C) in the tank, while its upper side serves as a support for placing the microgreens. A cabin of acrylic with an exhaust/ventilation system (CHIPSCE, 075‐1212), with a speed of 1500 rpm, was coupled above the reservoir to promote forced convection above the microgreens.

The microgreens were spread over the heated support using a doctor blade with 2–3 mm of thickness. The foam and support surface temperature were measured using an infrared thermometer (AKSO, AK30 new) with a foam emissivity of 0.96 (Incropera et al. 2007) and polyester film emissivity of 0.92 (Durigon et al. 2018). The temperature distribution in the thin layer was measured at three points, equidistant at 10 cm intervals, and 5 cm away from the edges. The temperature and relative humidity inside the cabin were measured using a thermo‐hygrometer (Incoterm).

#### 2.4.2 Freeze‐Drying

The foam was placed in glass jars and initially frozen in an ultra‐freezer (SANYO, MDF‐U73VC) at −75°C ± 5°C for 40 min. The samples were dehydrated in a freeze dryer (CHRIST, Alpha 1‐2 LDplus) under a vacuum pressure of 0.014 mbar at −58°C for 48 h.

#### 2.4.3 Air Circulation Oven (OD)

The foam was placed on silicone mats, with a thickness of 2–3 mm, and dehydrated in an air‐circulating oven (TECNAL, TE‐394/2) at 70°C for 2.5 h. The temperature evolution of the foam was measured using an infrared thermometer (AKSO, AK30 new) with a pulp emissivity of 0.96 (Incropera et al. 2007).

### 2.5 Physicochemical Properties

#### 2.5.1 Moisture and Water Activity

The moisture content of powder was determined using infrared radiation drying (105°C) in an analyzer balance (SHIMADZU, MOC63u). The water activity was determined using an analyzer for water activity (AQUALAB, CX‐2) at 25°C.

#### 2.5.2 Color

The color was determined on the CIELAB scale using a Color Muse colorimeter, and the values of lightness (*L* ^*^), chromaticity (*a* ^*^) and (*b* ^*^), chroma (*C* ^*^), and hue angle (*h*°).

#### 2.5.3 Hygroscopicity

The powder mass (0.1–0.2 g) was placed in hermetic container with a saturated solution of sodium chloride (NaCl) to maintain the relative humidity of the environment at 70% (Tonon et al. 2008). After 7 days, the mass of the samples was measured, and hygroscopicity was expressed as g of moisture adsorbed per 100 g of dry sample mass (g/100 g).

#### 2.5.4 Anthocyanins

The powder (0.2 g of each sample) was stored in Falcon tubes with 20 mL of 95% ethanol extraction solution with 1.5 N HCl (85:15 v/v). The tubes were shaken and allowed to stand for 12 h at 5°C in the dark. Subsequently, the samples were centrifuged (EPPENDORF, 5804R) and filtered. After a 2 h resting period in the dark at room temperature, anthocyanin readings were taken using a spectrophotometer (Jenway, 6705 UV/VIS) at a wavelength of 535 nm (Lees and Francis 1972). The results were expressed in mg of anthocyanin per 100 g of dry weight.

#### 2.5.5 Extraction

The extraction for analysis of total phenolics, total flavonoids, and the AA was performed using one gram of each powder, which was added to 15 mL of 80% ethanol. The extracts were centrifuged (EPPENDORF, model 5810 R) at 25°C for 15 min and at 12,000 rpm.

#### 2.5.6 Bioactive Compounds Content

The total phenolic content (TPC) was determined using gallic acid (GAE) as the reference standard and a calibration curve (0.03–0.34 mg/L). For analysis, 0.5 mL of extract filtrate was mixed with 2.5 mL of 0.2 N Folin–Ciocalteu reagent solution and 2 mL of sodium carbonate. The solution was incubated at a 96‐well microplate spectrophotometer (SpectraMax‐M2, Molecular Devices, USA) for 90 min and read with a wavelength of 760 nm (Singleton and Rossi 1965).

The total flavonoid content (TFC) was determined using QE as the reference standard and a calibration curve (0.05–0.47 µM/mL). For analysis, 1.5 mL of extract filtrate was added with 1.5 mL of aluminum chlorate at 2% of methanol and the reading with a wavelength of 415 nm (Quettier‐Deleu et al. 2000).

#### 2.5.7 Free Radical Capture Activities (ABTS and FRAP)

The ABTS was determined and read with a wavelength of 734 nm (Rufino et al. 2006). The FRAP was determined and the reading with a wavelength of 595 nm (Rufino et al. 2006). Calibration curves were constructed using the analytical standard Trolox: ABTS (0–4000 µM/L) and FRAP (66–433 µM/L) (Rufino et al. 2006).

#### 2.5.8 Fourier Transform Infrared Spectroscopy (FTIR)

FTIR was used with Agilent Technologies equipment (Cary 630), equipped with a total attenuated reflectance (ATR) accessory with a diamond crystal. Absorption spectra in the infrared region (range 500–4000 cm^−1^) with a resolution of 4 cm^−1^ and 256 scans were obtained using the ATR technique (Pavia et al. 2001). FTIR analyses were performed on samples with a powder aspect, placing each sample (triplicate) on the surface of the equipment crystal for respective reading. The functional groups were identified according to Pavia et al. (2001), complemented by data available in scientific articles. This approach enabled accurate assignment of characteristic absorption bands in the plant material. The spectra were plotted and optimized using OriginPro 2022 v.9.9.0.225 (SR1) x64 software.

### 2.6 Statistical Analysis

Experimental data were analyzed using ANOVA and the Tukey test with a 5% error probability (*p* < 0.05) in Statistica 8.0 (StatSoft Inc., Tulsa, USA).

## 3 Results and Discussion

### 3.1 Foam Characterization

The foam is composed of maltodextrin, starch, and an emulsifier. Maltodextrin acts as a carrier and encapsulating agent. Starch contributes to foam structure and stability by increasing viscosity and water‐binding capacity. Emulsifier provides foaming and emulsifying properties. These components also protect against light, oxygen, and moisture, helping preserve product quality and flavor (Bu et al. 2021; Tonon et al. 2008).

**FIGURE 3:**
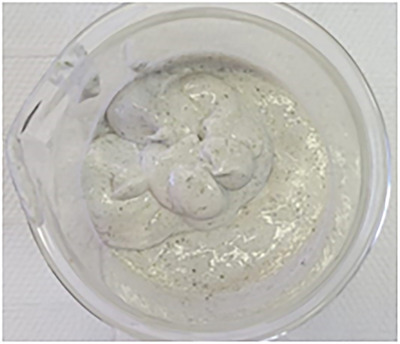
Cast‐tape drying experimental apparatus.

The foam showed 4 °Brix of soluble solids, moisture of 93.6% ± 0.28% (w.b.) and foam stability exceeding 92% over a 120 min analysis. The foam's whitish hue (Figure 3) comes from the white pre‐gelatinized starch, maltodextrin, and emulsifier, with slight green tones from chlorophyll in cabbage microgreens. However, the presence of anthocyanin, present in the hypocotyl of the red cabbage microgreen, is not observed. Anthocyanins exhibit pH‐dependent color changes in aqueous solutions. The fresh (in natura) red cabbage microgreens extract showed a pH of 5.83 ± 0.07, whereas the foam containing pre‐gelatinized starch, Emustab, maltodextrin, and red cabbage microgreens presented a pH of 5.96 ± 0.01. After drying, the average pH values of the powders were 6.00 ± 0.02 for FD, 5.50 ± 0.01 for CTD, and 5.70 ± 0.02 for OD. At pH 5.96, they become colorless, explaining the whitish hue of the formulated foam. Under extreme acidity (pH 1–2), they appear intensely reddish due to the prevalence of the flavylium cation form (AH+). As pH increases beyond 2, there is an equilibrium between the flavylium cation and a carbinol pseudo base. This shift leads to progressive color loss, resulting in near colorlessness at pH 6 (Giusti and Wrolstad 2001; Guimarães et al. 2012).

### 3.2 Cast‐Tape Drying

**FIGURE 4:**
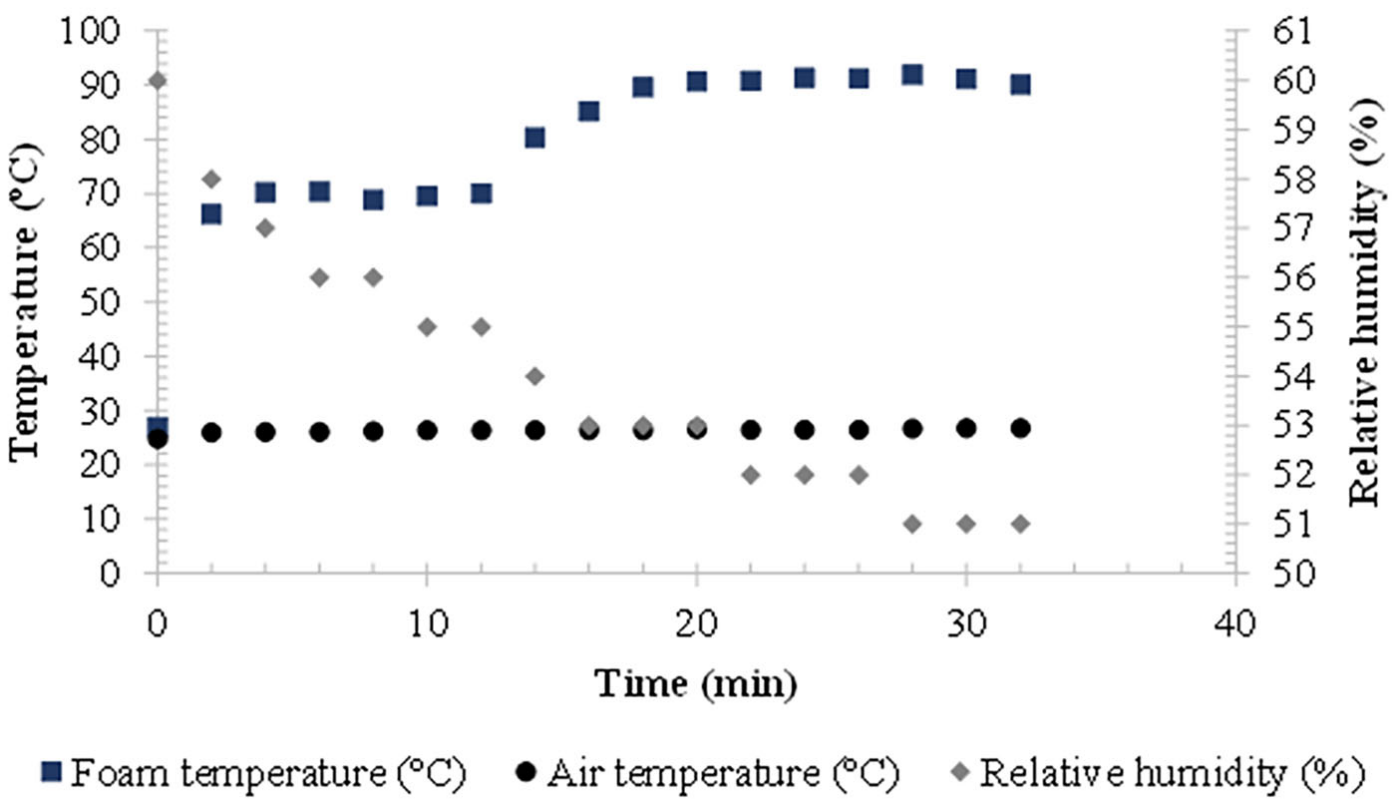
Temperature profile of microgreens of red cabbage foam, air temperature, and relative humidity during CTD process.

The final drying time in CTD was 32 min to achieve moisture below 0.066 ± 0.003 g/g (d.b.). The support temperature was 96°C ± 2°C, and during the constant rate, the foam microgreens temperature was between 66.1°C and 70.1°C (Figure 4). During this period (until the 12th‐min), dehydration was controlled by heat transfer fluxes from the water vapor. All the heat reaching the foam evaporates the water on its surface due to high initial moisture and thin pulp, resulting in negligible internal mass transfer resistance (Zotarelli et al. 2015; Durigon et al. 2018). The foam temperature stays constant and equal to the wet bulb temperature, about 26°C lower than the support temperature, due to evaporative cooling from air circulation over the foam. The foam temperature increased at the end of evaporation due to decreasing drying rate. The foam reached critical moisture, initiating capillarity and diffusion mechanisms for water vapor, which slowed water removal. During this period, drying depends on process conditions such as speed, temperature, and relative humidity of the air (Brooker et al. 1992). Additionally, heat transfer is not compensated by mass transfer because internal resistance to water transfer exceeds external resistance, causing an increase in product temperature. This behavior was also observed by Durigon et al. (2018) when dehydrating tomato juice (2 mm thick pulp and using hot water at 95°C as a heat source). Similarly, Da Silva et al. (2022) reported the same behavior when drying tucumã pulp by CTD using 2 mm thick pulp and a support temperature of 95.2°C ± 0.5°C.

As drying progresses, the air's relative humidity decreases gradually. Initially, water vapor is abundant as water evaporates from the foam's surface. As drying continues, vapor presence diminishes, reducing air saturation.

### 3.3 Air Circulation Oven

**FIGURE 5:**
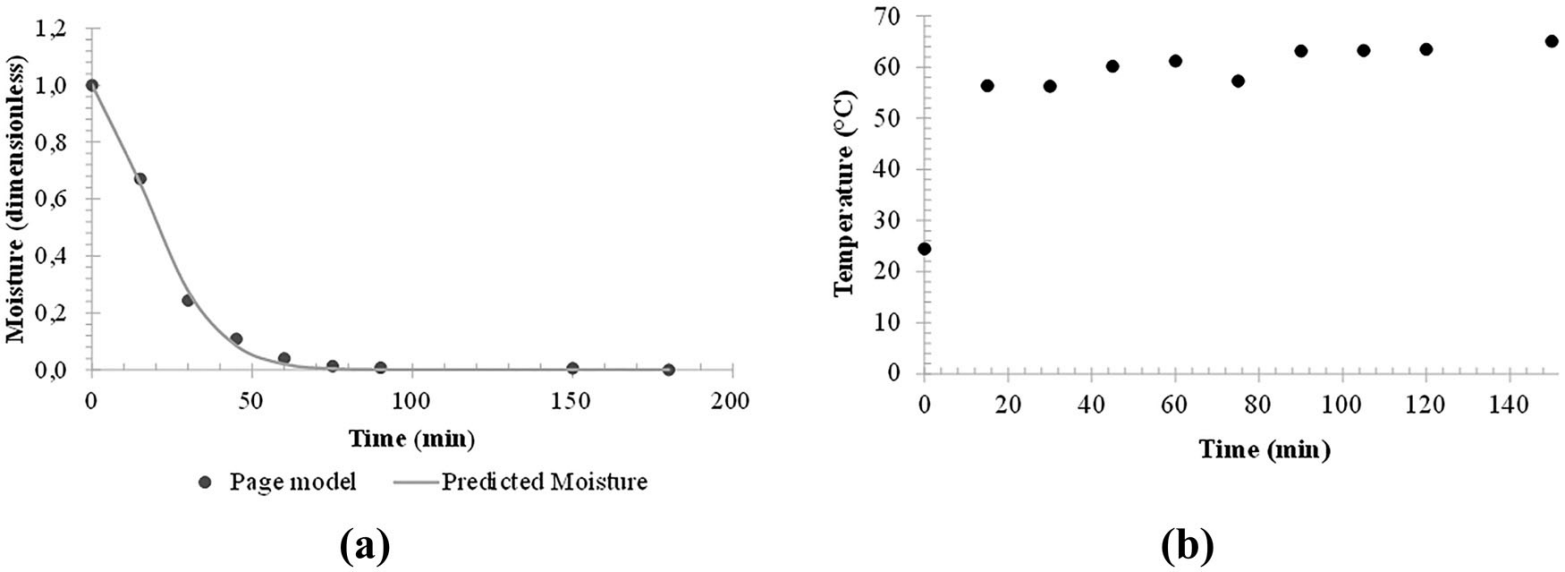
(a) Drying curves and (b) time‐temperature evolution of microgreens of red cabbage foam during air circulation oven at 70°C.

The drying time of the foam in OD was 150 min to achieve a moisture content of 0.078 ± 0.007 g/g (d.b.) (Figure 5). The exponential Page model was fitted (*R* ^2^ = 0.998, sum of squared error less than 0.002) to the experimental moisture data, with a drying constant (*k*) of 0.005 min^−1^ and the parameter *n* of 1.60. Ozcelik et al. (2024) reported that hybrid microwave–hot air drying (360 W, 60°C) of red cabbage juice foam. These authors reported reduced moisture from 93.47% to 8.62% and shortened drying time by more than 90% (20 min) compared with conventional hot air drying at 60°C (236 min). In the initial stages of the process, the foam is adapted with an increase in its temperature, reaching around 56°C, and remaining constant until 30 min (Figure 5). The decline in moisture exhibited linear behavior until 30 min, indicating a period of constant‐rate drying, justifying the foam temperature values. After, the foam temperature gradually rises, reaching close to 65°C at 150 min of drying. The increase in foam temperature is attributed to the decreasing drying rate, as internal resistance to water transfer limits mass transfer relative to heat transfer (Brooker et al. 1992).

### 3.4 Physicochemical Characterization of Microgreens Powder

The FD powder exhibited significantly higher moisture content and water activity values than the CTD and OD powder (Table 1). Moisture content values were below 0.1 g/g and depended on the sample's chemical composition, drying time, and air conditioning, such as temperature and relative humidity, for CTD and OD powders. Meanwhile, FD powder depends on the chemical composition of the sample and drying time.

**TABLE 1:** Moisture, water activity, hygroscopicity, and color parameters (*L* ^*^, *a* ^*^, and *b* ^*^) of microgreens red cabbage powder obtained by oven drying (OD), freeze‐drying (FD), and cast‐tape drying (CTD).

Components	OD	FD	CTD
Moisture (g/g)	0.078 ± 0.007^b^	0.097 ± 0.013^a^	0.066 ± 0.003^b^
Water activity	0.452 ± 0.044^c^	0.714 ± 0.001^a^	0.538 ± 0.016^b^
Hygroscopicity (%)	10.34 ± 0.99^a^	6.87 ± 1.48^b^	11.89 ± 1.14^a^
*L* ^*^	53.8 ± 6.8^b^	69.3 ± 7.6^a^	48.6 ± 6.1^b^
*a* ^*^	4.5 ± 2.4^a^	−1.5 ± 1.1^b^	4.8 ± 0.9^a^
*b* ^*^	10.7 ± 3.3^ab^	8.4 ± 1.6^b^	12.2 ± 1.4^a^
*C*	11.9 ± 2.6^a^	8.7 ± 1.7^b^	13.2 ± 9.7^a^
*h*°	65.2 ± 15.5^b^	94.3 ± 15.8^a^	68.3 ± 4.7^b^

*Note*: The values are the mean of three replicates ± standard deviation. Means with the same superscript letters (a–c) within a line indicate no significant differences according to Tukey's test (*p* ≤ 0.05).

The water activity values of CTD and OD powders are considered suitable for preserving the quality of the product (<0.6) (Rahman 2009). However, the FD powder exhibited a higher moisture content and water activity value <0.7 (Table 1). Due to technical limitations, FD time was fixed at 48 h, and microgreens were characterized under these conditions to ensure experimental consistency. Extending drying time or adjusting process conditions could further reduce water activity. Thus, the observed water activity highlights the need for process optimization or complementary stabilization strategies to ensure product safety and storage stability.

The hygroscopicity of all powders was below 12% (Table 1), indicating that they are not sticky in environments with a relative humidity of 75%. The FD powder is the least susceptible to moisture absorption compared to other samples. Low hygroscopicity ensures chemical and microbiological stability during storage (I. Tontul et al. 2018).

**FIGURE 6:**
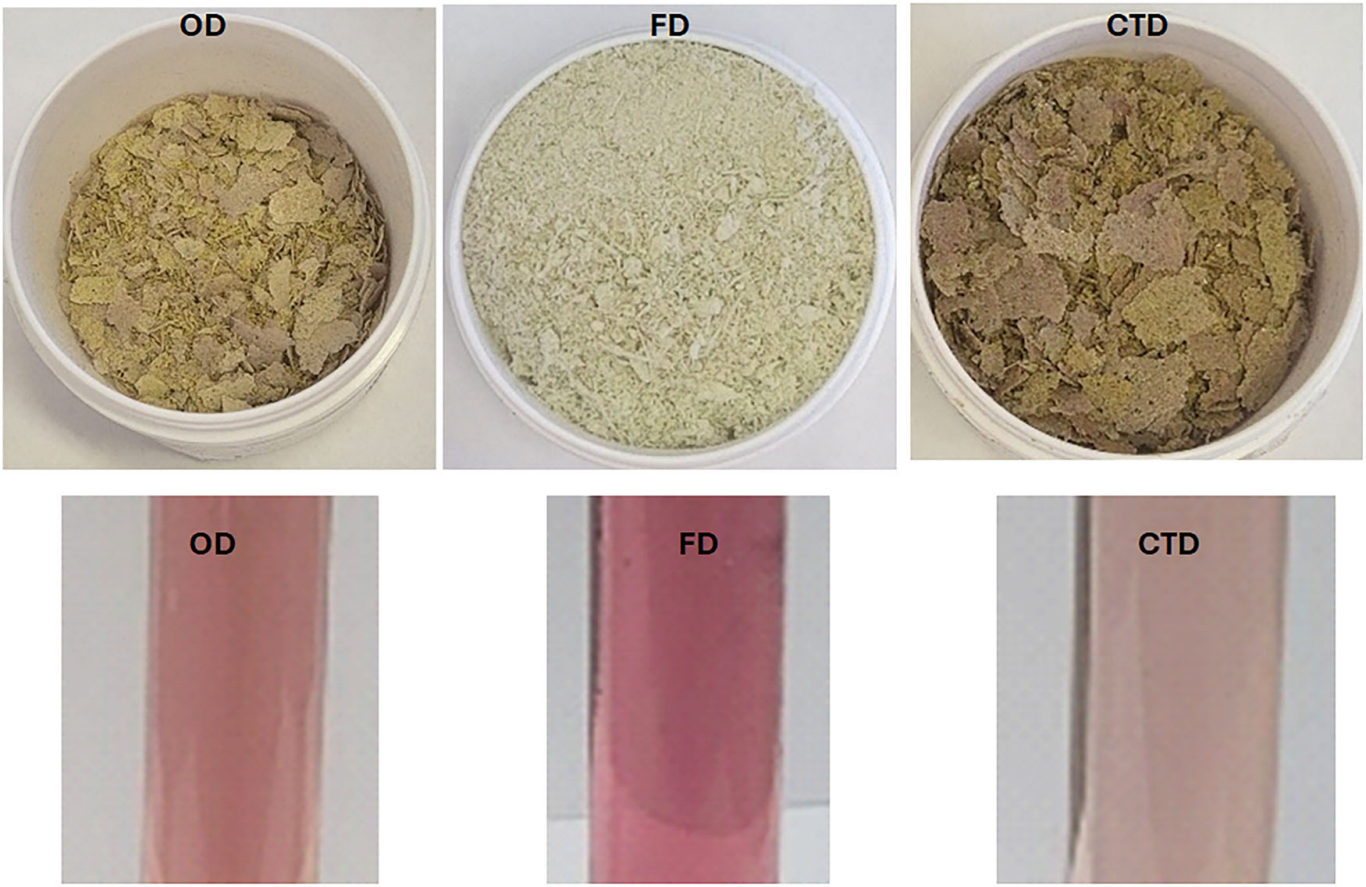
Photographic Images of microgreens of red cabbage powder obtained by OD, FD, and CTD. Extract of microgreens of red cabbage powder obtained by FD, OD, and CTD for anthocyanin determination. CTD, cast‐tape drying; FD, freeze‐drying; OD, oven drying.

The FD powder showed a significantly higher value for brightness (*L* ^*^) (Table 1). Brightness is associated with lightness, and higher values may be linked to its whitish hue, as shown in Figure 6. The brightness values showed no statistical difference between CTD and OD powders (Table 1).

The *a* ^*^ values showed no statistical differences between OD and CTD powders. However, the FD powder exhibited a greenish hue (−*a* ^*^) (Table 1). Red cabbage exhibits purplish‐red hypocotyls due to anthocyanin accumulation (Kowitcharoen et al. 2021). The characteristic purple color of red cabbage microgreen hypocotyls was not predominant in the powders, particularly in the FD sample (Figure 6), like what was observed for the foam. This color loss is attributed to the pH of the medium (5.96 ± 0.01) and the powder pH values (6.00 ± 0.02 for FD, 5.50 ± 0.01 for CTD, and 5.70 ± 0.02 for OD), as discussed earlier. The anthocyanin compounds are unstable during processing and storage and can undergo degradation or alteration due to temperature, pH, oxygen, and light (Chitarra and Chitarra 2005).

The *b* ^*^ (yellowness) values showed no statistical differences between the OD and FD powders. Additionally, the OD powder had a *b* ^*^ value significantly equal to the CTD powder (Table 1). The increase in temperatures can lead to the replacement of magnesium in the chlorophyll structure by hydrogen, resulting in the conversion of chlorophyll into pheophytins (Rudra et al. 2008). This transformation imparts a more yellowish/brownish hue to the sample. This explanation elucidates why the *b* ^*^ parameter related to coloration is higher in samples subjected to thermal processes with heat application (OD and CTD).

The chromaticity (*C* ^*^) values were higher for those obtained by OD and CTD and showed no statistical differences, indicating higher saturation (Table 1). The hue angle (*h*°) for the OD and CTD powders showed angles smaller than 90°, close to the orange tone, whereas the FD powder had an angle greater than 90°, indicating a hue more toward yellowish green (Table 1). A variation in hue from purple/red to green in the samples is attributed to the characteristics of red cabbage microgreens (Kowitcharoen et al. 2021). Anthocyanins are the most common group of pigmented flavonoids responsible for plants’ red, pink, purple, and blue colors (Taiz and Zeiger 2013). Meanwhile, chlorophylls are the most abundant green pigments in plants, algae, and bacteria (Petterson et al. 2022).

Figure 6 illustrates the extracts used in the anthocyanin analysis, where the powders were immersed in an acidified ethanol solution (pH of 1.5). These extracts exhibit a coloration distinct from that observed in the microgreen foam and powders (Figures 3 and 6). This indicates that due to the reversible reaction, pH influenced the hue of the powders and the foam. In an acidic medium (pH between 1 and 2), anthocyanins display intensely reddish coloration due to the presence of the flavylium cation (Giusti and Wrolstad 2001). Thus, the FD extract shows a more intense color, followed by OD and CTD extracts.

The phenolic compounds or polyphenols are secondary metabolites plants produce with important antioxidant function (Zagoskina et al. 2023). These compounds are divided into two categories, namely, flavonoids (anthocyanins and quercetin) and non‐flavonoids (cinnamic acid, benzoic acid, proanthocyanidins, stilbenes, coumarins, lignans, and lignin) (Hollman 2001). To better understand the effect of drying processes, TPC, TFC, anthocyanins, and AA of OD, FD, and CTD microgreens powder were determined (Table 2).

**TABLE 2:** Total phenolic, total flavonoids, anthocyanins, and antioxidant activity (AA) of microgreens red cabbage powder obtained by oven drying (OD), freeze‐drying (FD), and cast‐tape drying (CTD).

Process	Total Phenolic (mg GAE/100 g w.b.)	Total flavonoids (mg QE/100 g w.b.)	Anthocyanins (mg/100 g w.b.)	AA (FRAP) (µmol/100 g w.b.)	AA (ABTS) (µmol TE/100 g w.b.)
**OD**	48.2 ± 4.3^b^	282.6 ± 29.6^b^	480 ± 18^b^	571.0 ± 28.8^b^	2167.9 ± 62.8^b^
**FD**	98.5 ± 9.8^a^	455.7 ± 52.9^a^	611.4 ± 15.3^a^	2589.3 ± 76.5^a^	12,093 ± 1010^a^
**CTD**	35.5 ± 5.5^c^	187.6 ± 27.2^c^	273.2 ± 18.9^c^	446.8 ± 31.4^c^	1933 ± 143^b^

*Note*: The values are the mean of three replicates ± standard deviation. Means with the same superscript letters (a–c) within a column indicate no significant differences according to Tukey's test (*p* ≤ 0.05).

Abbreviation: AA, antioxidant activity.

FD resulted in a dried product with higher TPC than OD and CTD. The TPC in the OD powder sample showed a reduction of 51%, whereas the TPC in the CTD powder sample decreased by 64%. This decrease in TPC is likely due to the irreversible degradation of phenolic compounds caused by prolonged exposure to high temperatures (Guiné et al. 2015). The thermal degradation kinetics of total phenolics varies among different characteristics of the raw materials, primarily due to differences in phenolic compositions and the thermal stability of individual compounds (Kumar et al. 2022).

The TFC in the FD powder was significantly higher than that of the OD and CTD powder. This indicates that the high temperatures used during drying led to the degradation of flavonoids in red cabbage microgreens.

The FD process effectively preserves bioactive compounds by maintaining lower temperatures and reducing oxygen concentrations. Additionally, in the FD process, the formation of ice crystals within the matrix disrupts cell structures, facilitating the extraction of phenolic compounds by solvents (Shih et al. 2009).

Phenolic compounds and antioxidants are known to be volatile and thermally unstable. Prolonged exposure to heat and oxygen can induce irreversible changes in their chemical structure (Wu et al. 2019). In the OD process, the temperature ranges between 56°C and 65°C for 150 min. In contrast, during the CTD process, the temperature of microgreens foam reaches nearly 90°C from the 18th to the 32nd‐min of drying. Although the processing time is shorter in CTD than in OD, the higher temperature during CTD causes greater degradation of phenolic and flavonoid compounds. The extensive surface area of pulp exposed to air during the CTD and OD process likely plays a significant role in facilitating the oxidation reactions of hydroxyl groups, compared to FD (Wu et al. 2019). Oxygen exposure and light incidence were not controlled in the OD and CTD processes, which is a limitation of this study. During CTD, the exhaust and ventilation chamber is made of acrylic, allowing light to enter during drying. In contrast, the OD process does not allow light exposure. The higher light incidence, together with oxygen availability, may have contributed to the degradation of anthocyanin and phenolic compounds. These effects could not be separated from thermal effects. Future studies should control oxygen availability and light exposure to better clarify their individual roles in bioactive compound losses.

Total phenolic compound degradation during the drying of tucumã pulp by CTD was demonstrated (Da Silva et al. 2022). The more pronounced degradation of 39.5% occurred in decreasing drying period, when the pulp reached temperatures of 90°C. Additionally, a 17.5% degradation occurred during a constant rate period. These authors provide valuable insights on operating conditions and equipment design to reduce the degradation of bioactive compounds. Vacuum chambers, combined with drying processes, are advantageous in minimizing pulp temperature during experimentation (Da Silva et al. 2022) and can be tested in future studies with microgreens.

Anthocyanins are unstable when exposed to high temperatures or changes in pH (Vannuchi et al. 2022), and this instability is reflected in the total anthocyanin data (Table 2). The anthocyanin content of the microgreen powders differed significantly, indicating that the drying influenced their degradation. A higher quantity of anthocyanins was observed in the FD powder, followed by OD and CTD. The anthocyanins in the FD powder are more than double that observed in CTD, due to the temperature increased from the 18th‐min until the end (32 min) of the microgreens CTD drying, reaching almost 90°C. In addition to pH and heat, light is another significant factor affecting the color and stability of anthocyanins (Bobbio and Bobbio 2001). In the CTD process, the presence of light is more intense due to the exhaust and ventilation chamber being made of acrylic, which allows light transmission. Another factor to be observed is that the stability of anthocyanins to discoloration is considerably increased by the presence of phenolic acids (Bobbio and Bobbio 2001). Red cabbage microgreens are rich in phenolic acids; the literature reported 3461 mg of gallic acid equivalent/100 g d.b. (Tomas et al. 2021). The same effect is observed due to non‐anthocyanin flavonoids, such as QE present in red cabbage microgreens (De Freitas 2020). De Freitas (2020) reported anthocyanin values for FD red cabbage microgreens ranging from 190 to 228 mg/100 g in the cotyledon and 252 to 290 mg/100 g in the hypocotyl. These values are close to those observed for the powder obtained by CTD.

Phenolic compounds play a crucial role in antioxidant action, both directly and indirectly, by donating electrons to oxidant species, scavenging free radicals, chelating metal ions, and reducing the accumulation of reactive oxygen species. They also enhance the activity of antioxidant enzymes while inhibiting enzymes that promote pro‐oxidant effects (Hollman 2001). Changes in the antioxidant potential of red cabbage microgreens subjected to different drying were investigated using in vitro tests with FRAP and ABTS assays. The results indicate that the AA data align with the behavior observed for total phenolic compounds (Table 2). The FD sample maintained higher antioxidant activity, as determined by the FRAP assay, than OD and CTD samples, which showed significant reductions. The AA values for the CTD and OD powders, as determined by the ABTS assay, did not show significant differences; however, they were substantially lower than the values observed for the FD sample. Our findings are consistent with previous studies indicating that antioxidants in plant materials are susceptible to thermal drying processes (Kittibunchakul et al. 2023).

**FIGURE 7:**
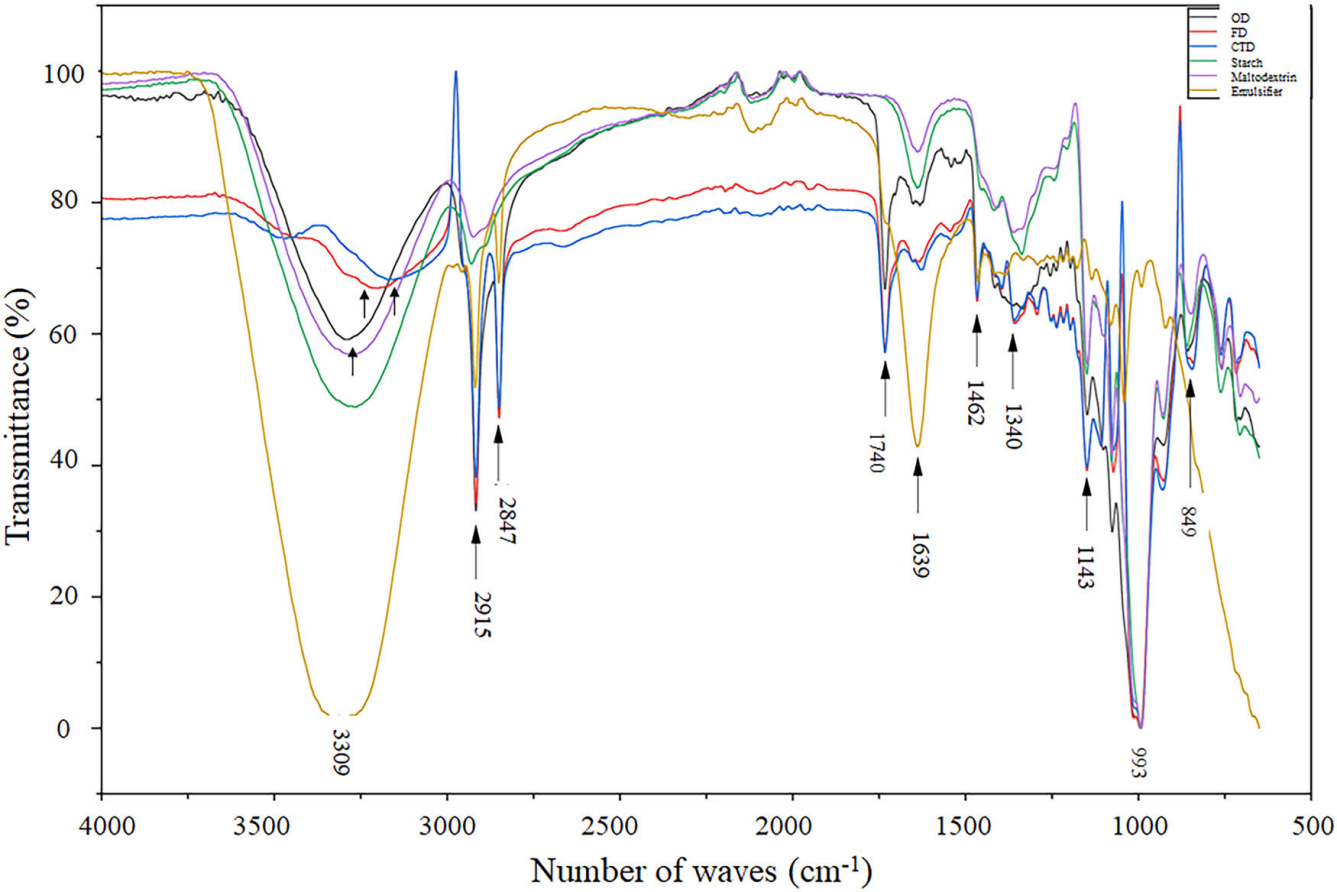
FTIR spectra of microgreens of red cabbage powder obtained by FD, OD, and CTD.

The FTIR profile of the general spectrum of microgreens red cabbage powder under different drying was similar, especially in FD and CTD powders, except for some of the identified bands in OD powders (Figure 7). The red cabbage microgreen powder should be considered a complex sample due to its production involving a mixture of red cabbage microgreens and adjuvants, which is rich in carbohydrates, proteins, fatty acids, fibers, phenolic compounds, vitamins, and minerals. The broadband at 3309 cm^−1^ corresponds to the OH stretching vibration, primarily in water molecules bound within the sample. It is more pronounced in the emulsifier sample due to the higher water content in this pure compound. A sharp peak in wave number 3296 cm^−1^ was observed for the OD powder, and less pronounced peaks in this band were 3173 cm^−1^ (CTD) and 3194 cm^−1^ (FD). This wavenumber represents the tensile O―H vibrations of hydroxyl groups as well as intramolecular hydrogen bond vibrations, mainly due to cellulose and hemicellulose, as suggested by Xie et al. (2017). All samples (CTD, FD, and OD) exhibited a band of saturated hydrocarbon groups at 2915 and 2847 cm^−1^. This band corresponds to the stretching vibration of C―H_2_, and nC―H_3_ identifies aromatic compounds with phenyl bonds typical of flavonoid compounds (Pereira et al. 2015). The band at 1740 cm^−1^ was associated with C═O stretching vibrations of esterified carboxyl groups in fatty acids. The band around 1600 cm^−1^ is attributed to the skeletal stretching vibration of the aromatic rings A and B flavonoids and the functional group ═C―O―C of the C ring of flavones (Różyło et al. 2021). The absorption peak at 1462 cm^−1^ was assigned to the C═C stretching vibrations of the aromatic ring in flavonoids and the bending vibrations of the CH_2_ and CH_3_ aliphatic groups in lipids (Simão et al. 2024). The band between 1300 and 880 cm^−1^ mainly results from the stretching vibrations of C―C and C―O bonds, and the angular C―O―H bonds of carbohydrates like starch (Türker‐Kaya and Huck 2017). The absorption peak at 993–1019 cm^−1^ was attributed to the pyran ring's stretching vibration and observed in pure maltodextrin and starch (Chen et al. 2023). The absorption peak at 849 cm^−1^ was assigned to the C―O―C stretching of glycosidic bonds presented in pure maltodextrin (Różyło et al. 2021).

Thus, the physicochemical and bioactive results may have been influenced by the foam‐forming agents (Mehrali et al. 2025). The inclusion of maltodextrin, pre‐gelatinized starch, and an emulsifier increases the proportion of inert matrix relative to the microgreen bioactives, leading to a dilution effect that lowers apparent concentrations of phenolics, flavonoids, and anthocyanins when expressed per unit mass of powder. Carrier materials also tend to dominate the FTIR spectra, as their carbohydrate‐related vibrational bands can overlap with or mask signals from phenolic and anthocyanin functional groups, particularly when these are present at lower relative abundance. Additionally, physical interactions between wall polysaccharides and bioactive compounds may affect extraction efficiency and spectral signatures (Mehrali et al. 2025; Ketnawa et al. 2018). Therefore, the reported physicochemical and bioactive properties should be interpreted as representative of the formulated powder system rather than of red cabbage microgreens alone.

Foam formulation was not optimized in this study and was maintained constant to allow comparison among drying methods. Nevertheless, the type and concentration of carrier and foaming agents may influence anthocyanin stability and color retention during thermal drying. Future studies should optimize foam formulation to improve bioactive preservation and assess long‐term powder stability under real conditions.

Despite its nutritional limitations, CTD offers technological advantages by reducing drying time through rapid foam spreading and high heat transfer, which benefits industrial productivity and scalability. However, increased exposure to heat, oxygen, and light leads to greater losses of phenolics, flavonoids, anthocyanins, and AA compared with FD and OD. Thus, CTD represents a trade‐off between processing efficiency and bioactive retention, favoring fast drying over maximum compound preservation.

## 4 Conclusion

The CTD process enables rapid drying of red cabbage microgreen foam, whereas OD and FD require longer times. Foam formulation allows CTD and OD powders to achieve low moisture, water activity, and hygroscopicity, whereas FD powder has higher water activity, requiring careful packaging and storage. The characteristic purple color of microgreen hypocotyls is less pronounced in powders, especially FD, but acidified ethanol treatment reveals higher color intensity in FD, followed by OD and CTD. FD, despite being time‐consuming and costly, ensures the highest retention of total phenolics, flavonoids, anthocyanins, and antioxidant activity. OD and CTD cause greater bioactive degradation due to temperature, oxygen, and light exposure. FTIR spectra confirm the presence of bioactive carbohydrates and fatty acids in all powders. Overall, FD is best for high‐value or fortified products. CTD offers rapid, industrially scalable drying at the cost of lower bioactive retention. OD provides a balance between efficiency and bioactive preservation. These findings guide the selection of drying methods, highlighting trade‐offs between processing efficiency and nutritional quality, and support the potential use of microgreen powders in food formulations. Future research should investigate the optimization of foam formulation and conduct long‐term stability studies, considering oxygen, light, and storage conditions, to maximize the preservation of bioactive compounds.

## Author Contributions

**Islaine De Jesus Silva**: data curation, methodology, investigation, validation, writing – original draft. **Mônica Silva De Jesus**: conceptualization, methodology, writing – review and editing. **Hannah Caroline Santos Araujo**: conceptualization, methodology, writing – review and editing. **Regina Santiago Campos Nascimento**: conceptualization, methodology, writing – review and editing. **Maria Terezinha Santos Leite Neta**: conceptualization, methodology, writing – review and editing. **João Paulo Natalino De Sá**: conceptualization, investigation, writing – review and editing, project administration. **Simone Mazzutti**: conceptualization, investigation, writing – review and editing, project administration, data curation. **Frederico Alberto De Oliveira**: conceptualization, methodology, investigation, funding acquisition, writing – review and editing, formal analysis, supervision, project administration. **Marcelo Augusto Gutierrez Carnelossi**: conceptualization, methodology, writing – review and editing. **Angelise Durigon**: conceptualization, investigation, funding acquisition, writing – original draft, methodology, validation, writing – review and editing, formal analysis, project administration, supervision, data curation, resources.

## Conflicts of Interest

The authors declare no conflicts of interest.
